# Reverse lateral upper arm flaps for treating large soft tissue defects extending from the elbow to the forearm

**DOI:** 10.1080/23320885.2022.2076683

**Published:** 2022-05-19

**Authors:** Hideki Okamoto, Yohei Kawaguchi, Shinji Miwa, Hisaki Aiba, Hiroya Senda, Satona Murakami, Kazuo Hayakawa, Yuji Joyo, Hideki Murakami, Hiroaki Kimura

**Affiliations:** a Department of Orthopaedic Surgery, Nagoya City University Graduate School of Medical Science, Nagoya City, Japan; b Department of Orthopaedic Surgery, Kanazawa University Graduate School of Medical Science, Kanazawa City, Japan; c Department of Rehabilitation, Nagoya City University Graduate School of Medical Science, Nagoya City, Japan

**Keywords:** Large soft tissue defect, elbow, tumor, trauma, reverse lateral upper arm flap

## Abstract

In this study, we report three cases wherein reverse lateral upper arm flaps were used to treat very large tissue defects extending from the elbow to the forearm. The flap sizes were 19 × 6.5 cm, 20 × 7 cm, and 17 × 7 cm. All flaps survived and elbow motion recovered sufficiently.

## Introduction

The reconstruction of soft tissue defects around the forearm and elbow can be challenging. Previous studies have reported several techniques for soft tissue reconstruction around the elbow. The reverse lateral upper arm flap is a useful and reliable treatment option for such defects because it facilitates the preservation of the major arteries [1,2]. The usefulness of reverse lateral upper arm flaps for elbow coverage has been reported, however, in most studies, these flaps have been used for small soft tissue defects in most studies [1–6]. In this study, for the first time, we report three cases wherein reverse lateral upper arm flaps were used to treat very large soft tissue defects extending from the elbow to the forearm after wide resection of a malignant tumor or severe trauma.

## Case report

This case series was approved by the appropriate institutional review board, and informed consent was obtained from all the patients. For this case series, cases treated with a reverse lateral upper arm flap between January 2012 and July 2015 were reviewed. Three patients (age range: 23–86 years) with very large tissue defects extending from the elbow to the forearm underwent flap implantation. In two patients, the defect was caused by wide resection of a malignant soft tissue tumor, while injury from a traffic accident caused the defect in the third patient. Patient data, including the flap sizes for each patient, are presented in Table 1.

**Table 1.** Patients’ data.

Case	Age, Sex	Diagnosis	Flap size/defect size	Donor site
1	61, M	Myxofibrosarcama	19 × 6.5 cm*/19 × 13 cm	Primary closure
2	23, F	Soft tissue defect by traffic accident	20 × 7.0 cm*/20 × 10 cm	Primary closure
3	86, M	Myxofibrosarcama	17 × 7.0 cm*/17 × 11 cm	Partially closure

*With skin bridge.

The flaps were designed along an axis from the deltoid insertion to the lateral epicondyle after Doppler ultrasound was used to confirm the course of the vessels. Dissection was performed with the patients in the supine position. A posterior incision was made before the flap was subfascially elevated to the intermuscular septum. The pedicle of the posterior collateral artery was easily identified within the septum and was divided proximally. Subsequently, an anterior incision was made, and the fascia was separated from the brachialis and brachioradialis, until the intermuscular septum was reached. The distal region of this flap was not separated to preserve the distal pedicle of the interosseous recurrent artery and vein. The reverse lateral upper arm flap was then transferred to the elbow and forearm with the elbow in a flexed position. The cast was fixed for 2 weeks; after the flap was settled, the patients performed elbow flexion and extension training exercises.

All the flaps survived completely with no complications. In all the patients, the defect size was larger than the flap size. The flap was placed at the center of the defect to ensure that the bone and nerve were covered. In two patients, skin grafts were placed around the flap, whereas negative pressure wound therapy was performed in one patient.

The donor site (width, 6.5–7.0 cm) was closed primarily in two patients. In one older patient (donor site width of 7.0 cm), the donor site was only partially closed, and a wet dressing was applied because the radial artery pulsation had disappeared after complete site closure.

### Individual cases

**Figure 1.**
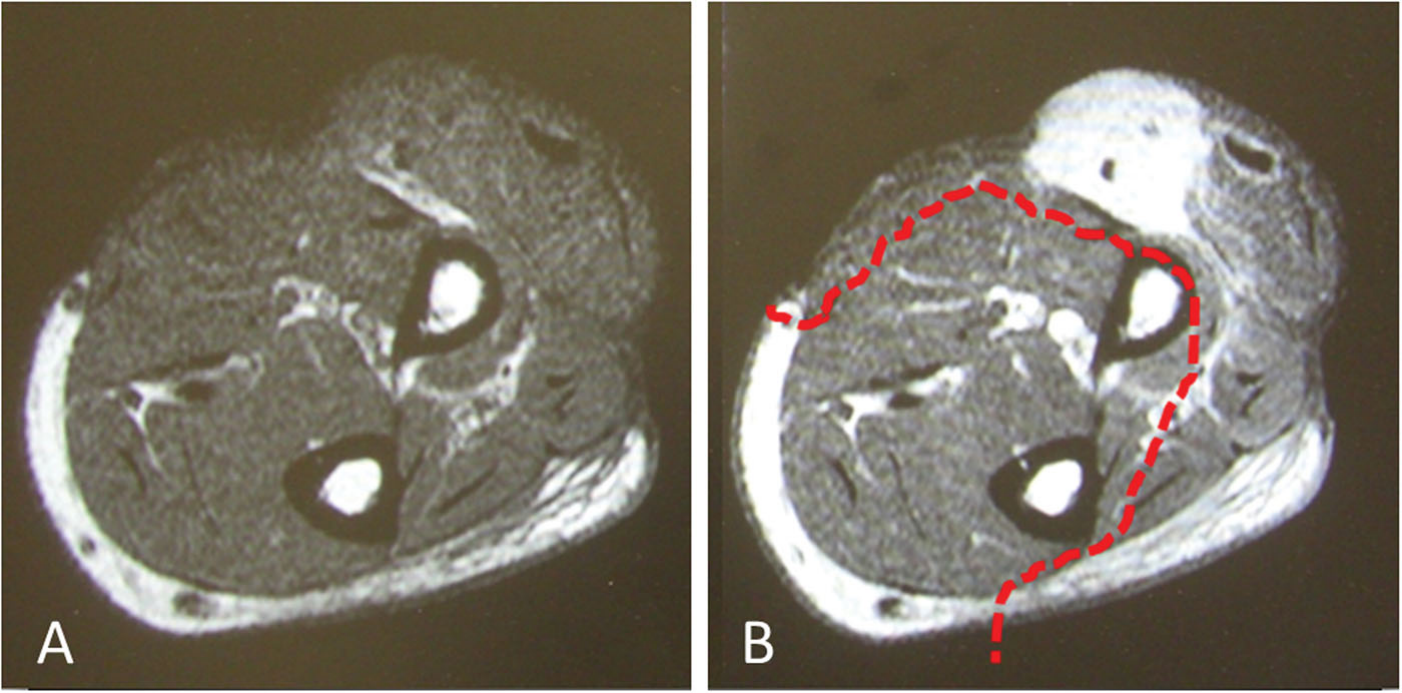
Images prior to reoperation. (A) MRI T1 weighted image. (B) MRI T1 weighted image with gadolinium enhancement. The dotted line indicates the extent of tumor resection. MRI: Magnetic resonance imaging.

**Figure 2.**
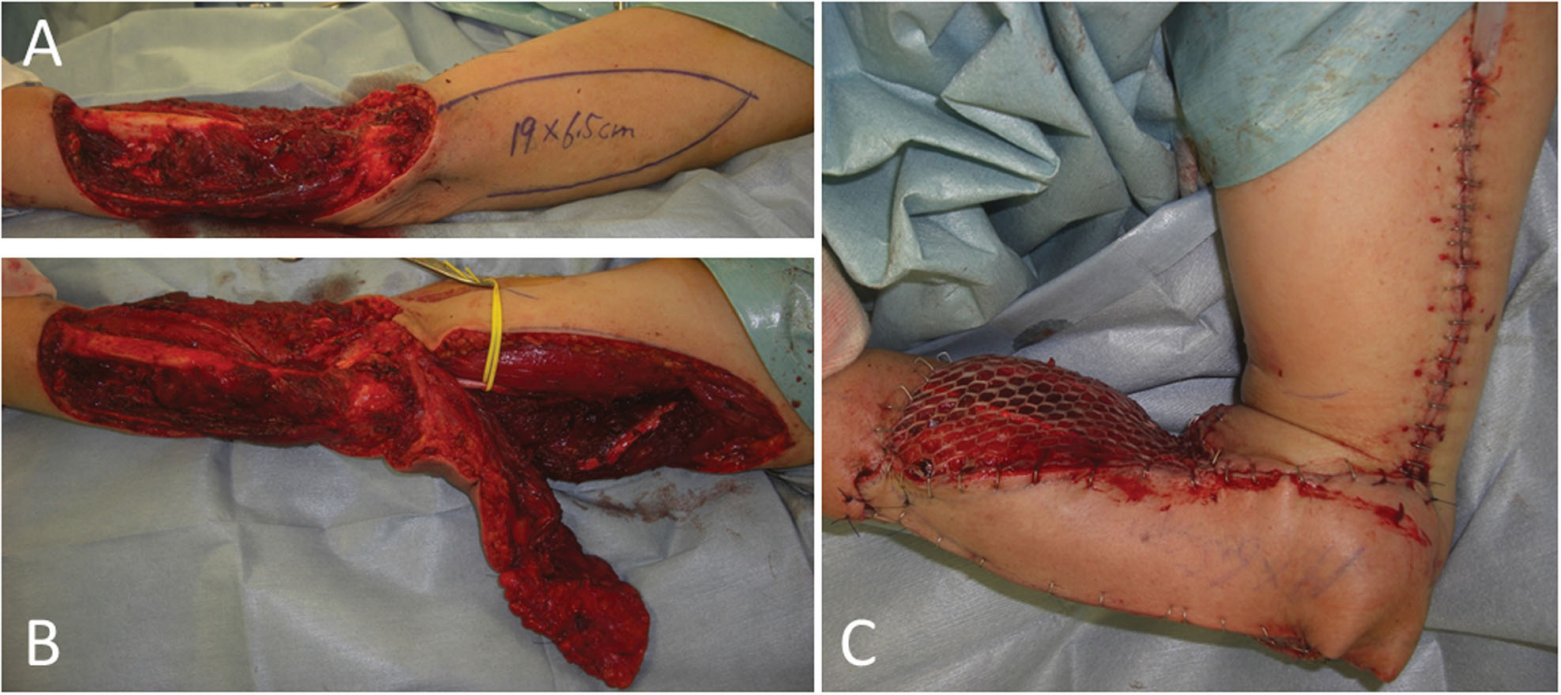
Intraoperative photographs of the patient in Case 1. (A) After extensive resection of the tumor, a large soft tissue defect, which exposed the radius, was observed. (B) While preserving the radial nerve, a 19 × 6.5 cm reverse lateral upper arm flap was raised. (C) The radius was covered with a flap, and a mesh skin graft was performed on a portion of the skin where the forearm muscle was exposed.

**Figure 3.**
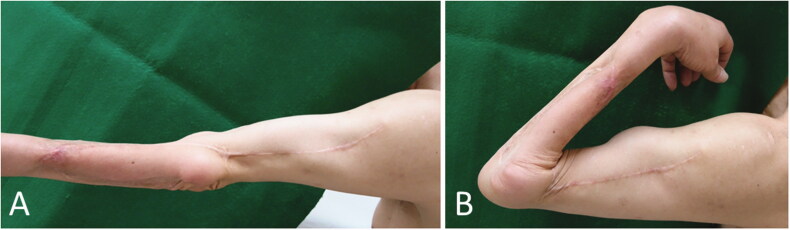
Case 1: Range of motion in the forearm and elbow 8 years and 3 months after the operation. (A) Degree of extension. (B) Degree of flexion.

Case 1: The patient was a 61-year-old man. After undergoing biopsy at a dermatology department in a general hospital, he was referred to our department for a left forearm tumor. After resection of the myxofibrosarcoma by open biopsy, wide resection and full-thickness skin grafting were performed. There was no range of motion limitation in elbow and forearm function. One year later, the tumor recurred, and the patient underwent a second surgery after neoadjuvant therapy with chemotherapy and radiotherapy (Figure 1). Wide resection was performed on the tissue defect, including the portion that had been skin grafted in the previous surgery. A 19 × 6.5 cm reverse lateral upper arm flap was implanted for extensive soft tissue defects (Figure 2). Eight years and three months postoperatively, the extension of the elbow was 0°, flexion was 140°, and International Society of Limb Salvage score was 27 points, which indicated adequate function preservation in the elbow and forearm (Figure 3).

**Figure 4.**
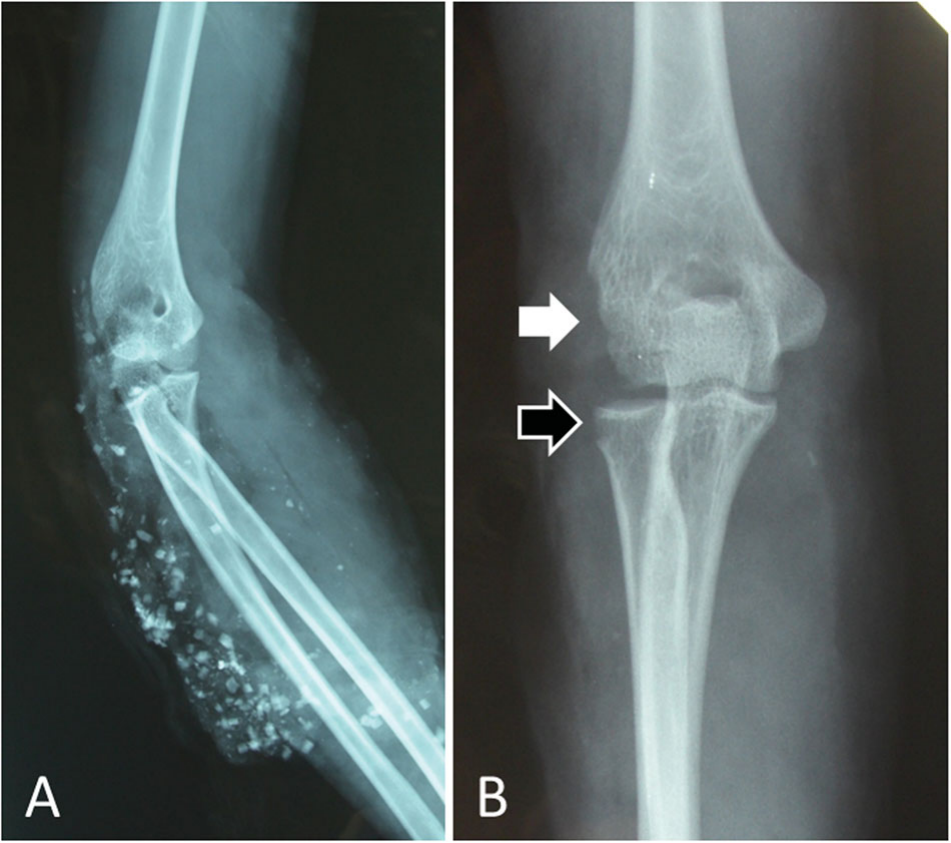
Radiographs of the patient in Case 2. (A) Radiograph taken immediately after the injury. The arm and elbow were contaminated with several windshield pieces. (B) Radiograph taken after debridement. The white arrow indicates deficient humeral condyles, while the black arrow indicates radial head defects.

**Figure 5.**
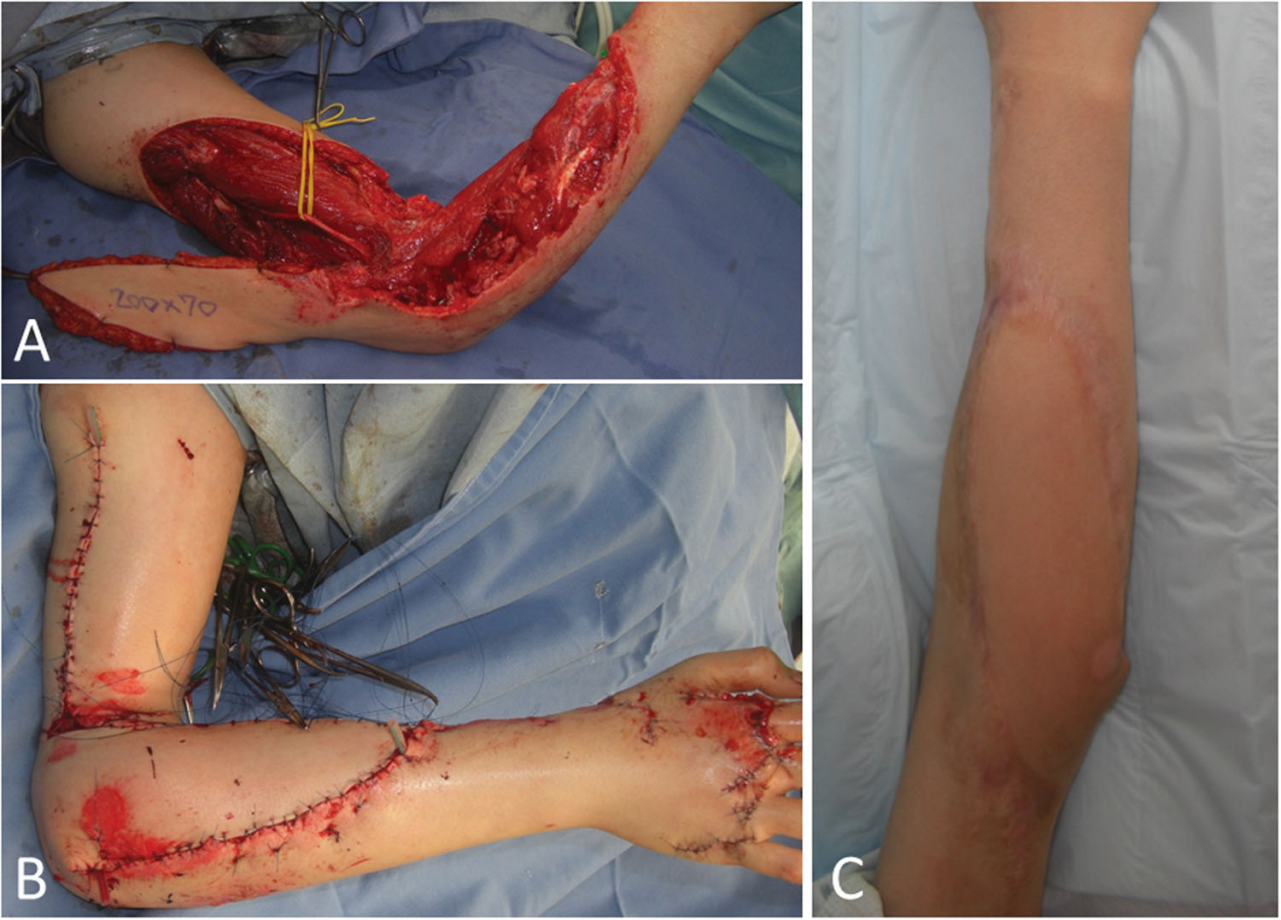
Intraoperative photographs of the patient in Case 2. (A) A 20 × 7 cm reverse lateral upper arm flap was designed. (B) The donor site was closed primarily. (C) One year and 5 months after flap implantation.

Case 2: The patient was a 23-year-old woman who was injured during a traffic accident. The wound was highly contaminated and was covered with an artificial dermis after debridement. Figure 4 shows an X-ray image obtained at the time of the injury. Two days after the injury, the extensive soft tissue defects were covered with a 20 × 7 cm reverse lateral upper arm flap (Figure 5). The flap survived, the extension and flexion of the elbow were 0° and 145°, respectively, and the patient returned to work 11 months after the surgery.

**Figure 6.**
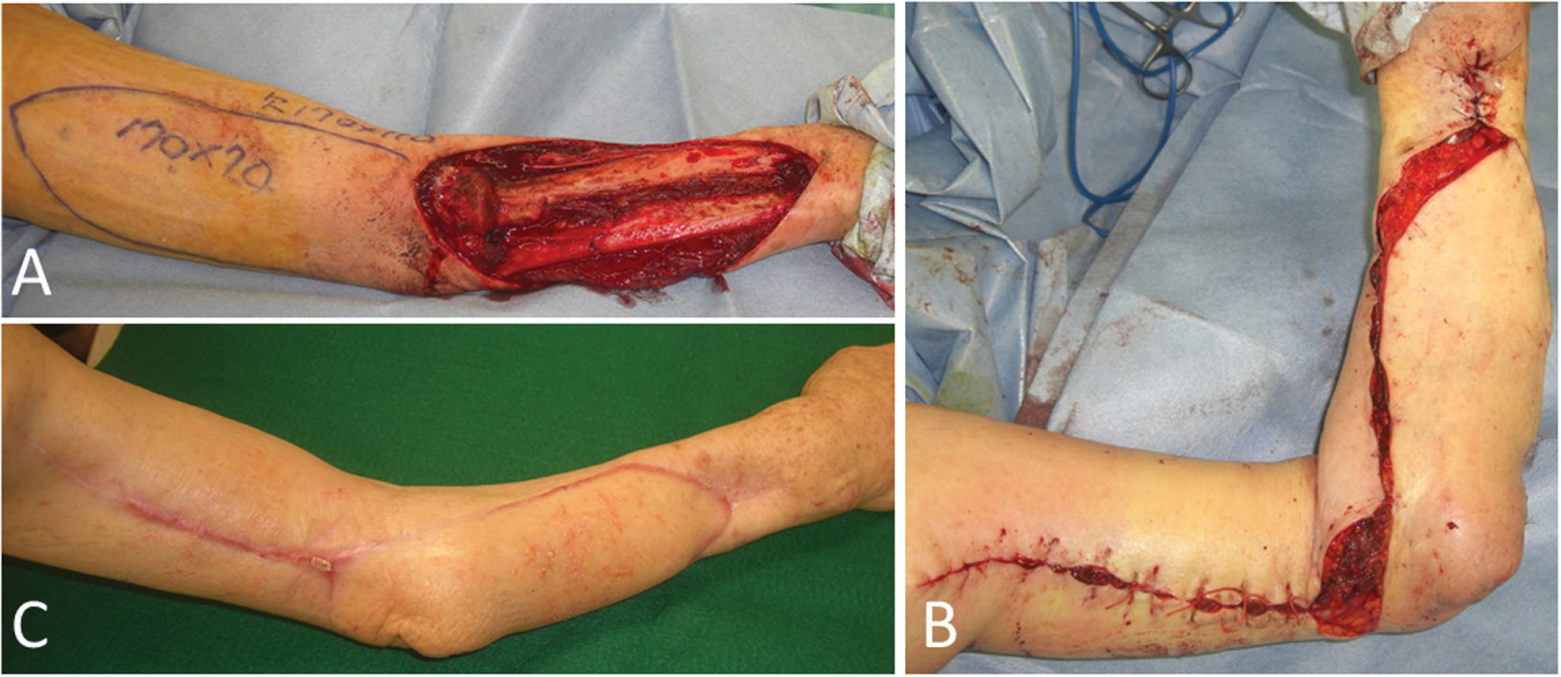
Intraoperative photographs of the patient in Case 3. (A) A 17 × 7 cm reverse lateral upper arm flap was designed. (B) The forearm and the elbow at the time of primary closure of the donor site. (C) Five months after flap implantation.

Case 3: The patent was an 86-year-old man with a myxofibrosarcoma in the right forearm, for which wide resection of the malignant soft tissue tumor and full-thickness skin grafting were performed. There was no range of motion limitation in elbow and forearm function. One year and nine months postoperatively, the tumor recurred locally, and a reoperation was performed. A 17 × 7 cm reverse lateral upper arm flap was implanted for the extensive soft tissue defects (Figure 6(a)). The donor site was temporarily sutured (Figure 6(b)); however, after suturing, the pulsation of the radial artery disappeared, and all fingers turned pale. Therefore, partial closure and negative pressure wound therapy were performed. Additional radiation therapy was provided after wound healing. Five months after the surgery, the healing process was uneventful (Figure 6(c)). Four years and four months after the operation, there was no recurrence of the tumor. The extension of the elbow was −20°, flexion was 125°, and International Society of Limb Salvage score was 26 points, which indicated adequate function preservation in the elbow and forearm.

## Discussion

Several treatment options exist for the coverage of tissue defects extending from the forearm to the elbow. The choice of flap coverage depends on a myriad of variables, including wound size, exposure to vital structures, comorbidities, and potential donor-site morbidities. Choices of flaps include local flaps, distant pedicle flaps, and free flaps. Local flaps are often too small, and their reliability is questionable. Radial forearm flaps are useful for the coverage of the elbow, however, these flaps are disadvantageous in that they sacrifice major blood vessels and require skin grafting at the donor site [7]. Choudry et al. [7] analyzed 99 patients who underwent soft-tissue coverage procedures for posterior elbow wounds; the radial forearm flap was the most frequently used. However, the authors explained that the main disadvantages of this flap included sacrifice of the radial artery, donor-site morbidities, and cosmesis. In 1986, Penteado et al. [8] were the first to report the use of posterior interosseous flaps to cover elbow defects. Posterior interosseous flaps also have many benefits, but can only cover small skin defects. Another common fasciocutaneous alternative is the lateral upper arm flap, which has the potential to reduce the occurrence of donor-site morbidities, rapidly restore elbow motion, and preserve major arteries [9]. The disadvantages of this flap include the possibility of sensory deficits in the posterior brachial cutaneous nerve distribution, and an unsightly scar on the lateral aspect of the arm [9]. However, for large skin defects that extend from the lateral side of the elbow to the dorsal side of the forearm, as seen in our study, the disadvantages of the lateral upper arm flap are not of significant concern because the skin defect is located in the sensory region of the posterior brachial cutaneous nerve.

When a lateral upper arm flap is taken, the anterior or posterior radial collateral arteries are raised as a pedicle. Song et al. [10] and Katsaros et al. [9] raised the anterior radial collateral artery and posterior radial collateral artery as pedicles, respectively. In 1986, Maruyama and Takeuchi [1] first reported a reverse lateral upper arm flap using a radial recurrent artery. Subsequently, Culbertson and Mutimer [2] described a reverse lateral upper arm flap using the interosseous recurrent artery. Martin et al. [11] and Casoli et al. [12] reported an extended lateral upper arm flap that extended up to the distal forearm and wrist. Morrison et al. [4] reported a two-stage reverse lateral upper arm flap based on the radial recurrent artery for coverage of complex traumatic elbow injuries. Herein, the authors elevated the flap and reinset it in its native position and sutured at the skin level. After at least 15 days, the flap was transferred to the elbow wound being treated. Ashfaq et al. [5] used at the reverse lateral upper arm flap to cover elbow defects caused due to burns. The flap sizes ranged from 9 × 5 cm to 15 × 6 cm, and the fasciocutaneous distal base was left intact [5]. In our cases, we implanted a reverse lateral upper arm flap using the interosseous recurrent artery. We succeeded in preserving the retrograde blood flow from the interosseous recurrent artery and covering large skin defects around the elbow and forearm by using a reverse lateral upper arm flap to create a pedicled flap, as described in the reports by Ashfaq et al. [5] and di Summa et al. [13], instead of an island flap. A skin bridge was maintained over the pedicle at the distal margin of the flap to improve the venous drainage in all cases.

Although free flap, such as the anterolateral thigh flaps, may be an alternative for elbow coverage, esthetics should be considered, especially for young women. In our report, the patient in Case 2 refused new wounds at a different site and requested that surgery be performed in the same extremity as the original wound. Thus, a reverse lateral upper arm flap was considered to be suitable for the patient. Ullah et al. described the experience of using the lateral upper arm flap both as a free flap and as a pedicled flap [6]. The average free lateral upper arm flap size was 12 × 6 cm (range: 7.5 × 5 cm–18 × 8 cm). All free lateral upper arm flaps were used for head and neck reconstruction. The average size of the pedicled lateral upper arm flap was as small as 8 × 5 cm.

In the lateral upper arm flap, a donor site width of 6.5 cm or less can be closed primarily and is cosmetically appealing. Ullah et al. reported that all donor sites were closed primarily, even if they were 8 cm in width, however, one patient experienced dehiscence secondary to necrosis at the wound edges because of closure under tension [6]. In our study, we were able to primarily close a donor site with a width of 7 cm without complication (Case 2); however, in Case 3, a 7-cm site could not be closed because of poor circulation to the periphery. This was believed to be due to the age related deterioration of the softness of the skin and tissues.

The limitation of this study was the small number of cases. The usefulness of procedure, and concurrently the data on larger flaps, depends on the physical attributes of the patient, larger flaps cannot be created in thin patients.

In conclusion, several flap variations can be used for small skin defects around the elbows and forearms, and these do not cause clinical inconvenience. However, in cases with much larger soft tissue defects extending from the elbow to the forearm, a reverse lateral upper arm flap should be considered for reconstruction after trauma or wide resection of a malignant tumor.
